# Validation of a Questionnaire to Assess the Perception of Women with Atopic Dermatitis in Family Planning

**DOI:** 10.3390/ijerph191710753

**Published:** 2022-08-29

**Authors:** Sara Alcantara-Luna, Ricardo Ruiz-Villaverde, Javier Domínguez-Cruz, Manuel Galán-Gutiérrez, Francisco Navarro-Triviño, Jose-Juan Pereyra-Rodriguez, Jose-Carlos Armario-Hita

**Affiliations:** 1 Dermatology Department, Hospital Universitario Virgen del Rocío, Avda. Manuel Siurot s/n, 41013 Sevilla, Spain; 2 Dermatology Department, Hospital Universitario San Cecilio, Avda. del Conocimiento s/n, 18016 Granda, Spain; 3 Dermatology Department, Hospital Universitario Reina Sofía, Avda. Menéndez Pidal s/n, 14004 Córdoba, Spain; 4 Dermatology Department, Hospital Universitario de Puerto Real, Calle Romería, 7, 11510 Puerto Real, Spain

**Keywords:** atopic dermatitis, gender, pregnancy, family planning, sexuality

## Abstract

Introduction: Atopic dermatitis (AD) is a highly frequent chronic inflammatory skin disease. It is important to know how women with AD approach family planning together with their disease. The aim of the present research is to develop and validate a questionnaire for women diagnosed with AD in order to measure their level of desire and gestational information. Materials and Methods: A multicenter cross-sectional study was conducted. Women between 18 and 45 years old with mild, moderate, and severe forms of the disease were included and disease-free controls. An exploratory factorial analysis of the primary components and varimax rotation was used to measure the validity of the construct. Cronbach’s α was used to measure the reliability of the individual scales and the global questionnaire. Results: In total, 150 valid questionnaires were included. The final questionnaire consisted of 23 items that converged on six factors. The six scales had adequate reliability: “Pregnancy” (Cronbach’s alpha = 0.95), “Conception” (Cronbach’s alpha = 0.93), “Concern-information” (Cronbach’s alpha = 0.82), “Breastfeeding” (Cronbach’s alpha = 0.81), “Sexual life” (Cronbach’s alpha = 0.79), and “Family planning” (Cronbach’s alpha = 0.67). The total Cronbach’s alpha of the questionnaire was 0.94. Discussion: This questionnaire is the first specific measurement instrument developed for women with AD of childbearing age that has demonstrated adequate levels of reliability and construct validity. We consider it useful and valuable to study aspects such as family planning in this patient profile, and that can influence their decision to have offspring.

## 1. Introduction

Atopic dermatitis (AD) is a highly frequent chronic inflammatory skin disease that can affect up to 4% of the population in our environment [1]. Prevalence is variable, reaching up to 10% of the general population, of which up to 15–30% have moderate/severe forms (10–20% in children and 1–5% in adults, especially in developed countries) [2]. Reports suggest that mild infantile forms disappear after puberty in up to 75% of cases. This prevalence is higher in women, although men predominate in childhood [3].

Many of the patients affected by moderate to severe AD are diagnosed at childbearing age or have continued their diagnosis since childhood, making them uncertain about the idea of having children and how pregnancy can affect the disease or how the disease can affect pregnancy [4]. Chronic inflammatory diseases, including AD, may experience a lower likelihood of pregnancy and a lower rate of live births compared to their healthy counterparts, and disease activity, comorbidities, and medication use can impact the probability of women becoming pregnant [5,6].

However, clinical trials usually exclude pregnant women. Although the actual use of certain drugs can sometimes suggest full-term pregnancies without complications, we do not have long-term safety studies on large series of pregnancies. Thus, one of the great challenges in the management of women of childbearing age is to provide recommendations on safe treatments. Sometimes the dilemma of achieving good control of the disease or completely eliminating the risk of maintaining treatment arises [7].

Dermatologists play an important role in providing information to the woman considering getting pregnant about the best treatment options and even planning the best time to do it [8]. Therefore, a “third party” comes to play a fundamental role in what, in theory, should be an important decision only for a couple. In this sense, it is essential to establish a good doctor–patient relationship, informing but taking into account that the final decision must be made by the patient. However, previous works have shown that most patients’ concerns regarding family planning and pregnancy (FPP) were inadequately or inconsistently addressed [9].

At the beginning of adult life, there are decisions about relationships and career paths, and thus, the cumulative impact on the life, social, emotional, and work of the patient can be substantial, with negative effects on the individual’s ability to reach their full potential. Each patient experiences the disease in one way, depending on the circumstances that occur at any given time, derived from the interaction between the burden of stigmatization, associated physical and psychological comorbidities, coping strategies, and external factors that can intervene, modulated by the personality styles of each patient. The objective of this study is to carry out and validate a questionnaire that would allow us to determine and measure all these little-known reasons until now and that may influence the decision of women with moderate to severe AD to have children.

## 2. Material and Methods

A multicenter cross-sectional study was conducted. Women between 18 and 45 years old with a previous diagnosis of atopic dermatitis at least 6 months before the visit were included, as well as controls from women of the same age range who attended for some other unrelated reason (for example, nevus control). The patients were consecutively included in each center. Patients with mild, moderate, and severe forms of the disease were included to ensure that the data were representative.

Data Collect. An ad hoc questionnaire was designed for data collection. An exhaustive review of the literature was performed by three dermatologists with experience in the development of questionnaires (JPR, JDC, and SAL), who specifically searched for the most appropriate dimensions to be included (fears, uncertainty, and doubts of patients regarding her possible pregnancy; lack of information about family planning and how her illness can impact pregnancy and its outcome, or how pregnancy can alter her illness, or the treatments received to control her illness). Furthermore, focus groups formed by dermatologists and AD patients were asked to check the literature findings and identify potential issues. Based on the previous results, a draft battery of 25 questions was generated. Each item was submitted for evaluation, following a Likert 5-point scale (strongly disagree, disagree, neither agree nor disagree, agree, and strongly agree).

In addition to the questionnaire, the following parameters were collected: sociodemographic (date of birth, sex, educational level, place of residence, marital status, number of pregnancies, and number of children); clinical (parts of the body affected by their AD, year of diagnosis, EASI, SCORAD, IGA, BSA, VAS pruritus, DLQI, and comorbidities); treatment received for atopic dermatitis (topical, oral corticosteroids, conventional systemic drugs, and biological systemic drugs).

The patients filled out the questionnaire anonymously and voluntarily on paper. The questionnaire collection period was extended from 1 September 2021 to 28 February 2022.

Sample size. In total, 125 questionnaires were required based on current recommendations in case and variable studies in factor analysis and multiple regression (5 questionnaires for each item) [10]. We increased this number by about 20% to allow for possible incomplete or invalid questionnaires and had a final sample size of 150 questionnaires.

Statistic analysis. To validate the questionnaire, a factorial analysis of the main components was assessed. This technique is used to analyze the interrelationships between a large number of variables and to explain these variables in terms of their common underlying dimensions. The goal is to condense the information into a smaller set of variables (factors) with minimal loss of information. Varimax rotation was used. Rotation facilitates interpretation of the extracted factors since the variables will have a correlation close to 1 with one of the factors and close to 0 with the rest of the factors. Varimax rotation is an orthogonal rotation method that minimizes the number of variables that have high loadings on each factor. The maximum number of convergence iterations was fixed at 25. The number of possible factors to extract was not limited. To assess the reliability of the scales, the Cronbach coefficient was calculated. The level of statistical significance was defined as 0.05. Data were collected and analyzed with the SPSS v22 statistical package (IBM Corp., Armonk, NY, USA). Finally, the estimated factorial scores for each subject in each of the factors obtained in the factorial solution were stored. A descriptive analysis of the data was performed, with mean ± SD for continuous variables and percentage for qualitative data. To contrast the differences between these variables and possible explanatory factors (such as the severity of AD), a univariate analysis was carried out using the chi-square test (categorical variables) and the t-Student test (numerical).

Finally, to compare the differences in the scores of each scale and the global questionnaire, the t-Student was used. For the multivariate analysis, a linear regression was performed using the “forward” method between the global value of the questionnaire and the variables.

Ethical aspects of the study. All patients signed an informed consent form prior to inclusion in the study. The patients were treated according to usual clinical practice with no intervention. The study was carried out in accordance with the Spanish Organic Law on Data Protection (OLPD), and data analysis was performed anonymously. The study protocol was approved by the local Research Ethics Committee.

## 3. Results

### 3.1. Demographic and Clinical Characteristics

A total of 150 questionnaires were collected. None were excluded due to completion errors or incompleteness, so a total of 150 questionnaires were analyzed. The demographic and clinical characteristics of the sample are shown in Table 1.

### 3.2. Questionnaire Validation

Construct validity

Questionnaire validation

Construct validity. Once the exploratory factor analysis (EFA) was performed, the following criteria were applied to remove the items of the initial questionnaire that presented poor measurements: communality below 0.04, factor weight below 0.4, and next factorial weight less than 0.15 [11]. In this way, two items were eliminated.

The final version of the questionnaire, both the one used for validation in Spanish and a translation in English, is shown in Table 2. A KMO value of 0.78 and a Bartlett sphericity test of 2257.28 (*p* < 0.001) indicates the convenience of performing an EFA to extract the factors. The model explains 83.13% of the total variance, with a determinant value < 0.001.

The 23 elements were distributed into six factors (subscales) (Table 3).

- Factor 1 (24.84% of the variance explained). This was named ‘Expectations related to pregnancy’ (‘Pregnancy’). This factor includes six items related to the expectations of having a pregnancy during the disease, as well as the fact that pregnancy can make the disease worse, as well as taking medication during pregnancy. This factor is the one that explains the most variance and reaches the highest value of Cronbach’s alpha (0.95).
- Factor 2 (14.32% of the variance explained). This was titled ‘Preparation for pregnancy (Conception)’. This factor includes five items related to those aspects that the woman can take into account before pregnancy and can condition the conception, such as preparation with the doctor of the best moment, the medications that can be taken and those that must be withdrawn, etc. It is the second factor that explains the most variance and also presents a Cronbach’s alpha greater than 0.90 (0.93).
- Factor 3 (12.83% of the variance explained). This was called “Concern-information” in relation to four items that addressed questions about the importance of having adequate information about having children (from the doctor and external sources) and how this information impacts the desire and active search to have children. It has an adequate alpha value (0.82). Factor 4 (11.12% of the variance explained). This was named ‘Breastfeeding’ by including two items that value the relationship between the disease and breastfeeding. A third item asks about concerns about caring for the child, which also becomes this factor, probably due to the great benefit that our society attributes to breastfeeding. Cronbach’s alpha is 0.81.
- Factor 5 (10.02% of the variance explained). This was titled “Sexual life” and included two items, one directly related to the affectation that AD produces in sexual life and another more generic (discomfort with the disease) that, in the context of the questionnaire, converges with this other item that forms this factor. Cronbach’s alpha is 0.79.
- Factor 6 (10.00% of the variance explained). This was called ‘Family planning” and includes three items related to family planning conditioned by treatments and dermatologist recommendations to decide on offspring. It is the factor that explains the least variance and the only one whose Cronbach’s alpha is less than 0.70 (0.67).

Scores and relationship between scores and sociodemographic and clinical variables. Table 4 shows the scores achieved for each item and scale in the global questionnaire, separated by the control group and AD. The average values of the different items varied between 2.13 and 4.31 in the group of patients and between 1.08 and 3.75 in the control group. The global score for the questionnaire ranged between 23 and 115 in the patient group and between 23 and 107 in the control group (the theoretical score according to the number of items and range was 23–115).

Table 5 shows the relationships between different scale scores, the global questionnaire, and sociodemographic and clinical variables in the group of patients with atopic dermatitis. To do this, the numerical variables have been dichotomized based on the mean values obtained on the scale (age and duration of the disease) or based on relevant cut-off points in the disease (EASI 7 defines a mild disease, DLQI less than or equal to 3 means a low impact on quality of life), etc.

Regarding the global score of the questionnaire, two variables showed statistical significance: having at least one child and current or previous systemic or biological treatment (showing significantly higher scores in both cases). In the multivariate analysis, these same two variables maintained statistical significance (Table S1). According to this finding, these two scales are the ones that show differences in most questionnaire factors (factors 1, 2, 3, 4, and 6 in the case of having previous children; and factors 3, 4, and 6 for the use of systemic or biological treatments).

If we consider the score for the different factors in isolation, factor 5 (sexual life) is the one that presents a greater number of variables with significant differences. Specifically, this factor presents higher scores in patients with more years of variable evolution, higher EASI, and worse DLQI scores. However, paradoxically, this scale is not associated with having at least one child and current or previous systemic or biological treatment, which is associated with the global score.

## 4. Discussion

Few studies have studied the expectations and concerns that women with have regarding fertility, pregnancy, and lactation [12]. Some of the treatments needed to control it may influence all these stages.

In the questionnaire applied, one of the main topics we may highlight is the fact that AD affects the sexual life of women of childbearing age [13,14]. The sexual life of patients with atopic dermatitis of longer evolution (>13 years) is significantly affected compared to patients with less evolution in time (<13 years) (6.00 ± 2.42 vs. 7.32 ± 2.80; *p* < 0.05). In women with a greater natural evolution of their AD, which covers their first years of sexual life, this can be altered in the long term. These data are consolidated when we observe that the sexual life of women with moderate to severe atopic dermatitis (EASI > 7 or DLQI > 3) was again significantly affected compared to patients with mild atopic dermatitis (EASI < 7 or DLQI < 3) (6.31 ± 2.71 vs. 8.00 ± 1.07; *p* < 0.05 and 5.70 ± 2.82 vs. 7.40 ± 2.09 *p* = 0.05, respectively).

Regarding pregnancy, unlike other dermatoses, it worsens AD in 50% of women who suffer from it, although these data are based on studies carried out in the early 1990s. From a pathophysiological point of view, mutations in the epidermal filaggrin (FLG) gene have been confirmed to determine a greater number of AD outbreaks during the gestational period [15]. We also cannot forget the influence of hormonal factors inherent to pregnancy itself. Cho et al. [16] in 2010 reported that 46% of pregnant women with AD had experienced deterioration in their baseline status during pregnancy. Interestingly, no changes in IgE and EASI levels had been demonstrated between women with and without flares.

Two variables have shown differences in the global score of the questionnaire: the fact of being a mother and the use of systemic or biological treatments. Although a priori some confounder variables might be thought to act on them, such as severity or the longest time of evolution of the disease, multivariate analysis shows that they are the variables that have the most influence. In our research, being a mother seems to negatively and significantly affect all the dimensions we have explored. In five of six dimensions evaluated, pregnancy (21.19 ± 7.11 vs. 26.60 ± 3.55; *p* > 0.05) conception (16.74 ± 5.62 vs. 21.40 ± 4.22; *p* < 0.05) concern-information (9.46 ± 3.61 vs. 13.67 ±3.04; *p* > 0.05), breastfeeding (5.83 ± 2.69 vs. 7.54 ± 3.64; *p* > 0.05), and family planning (6.88 ± 2.54 vs. 10.12 ± 2.27; *p* > 0.05) are significantly affected in patients with AD. Motherhood must be quite complex, having to combine illness and motherhood per se.

Concerning the possible events that can happen during pregnancy, the Danish registry cohort exposed by Hamman et al. [17] has studied complications and the evolution of treatment in women with AD. AD has been found to be inversely related to the development of gestational diabetes but directly related to premature membrane rupture and staphylococcal neonatal septicemia. However, no relationship has been found with greater development of preeclampsia, prematurity, or non-staphylococcal neonatal septicemia.

An important point of view to consider are concerns that arise regarding the treatment that can be applied at this stage of life. It has been shown that the consumption of drugs, both topical and systemic, is lower at this stage of a woman’s life. The causes, however, are not well determined and could be attributed to an improvement in dermatosis or to the concern the woman has about the effect of these treatments on the fetus. Patients who are in systemic treatment have a greater affectation on the dimension of “Concern-information” in a statistically significant way compared to patients who are only in topical treatment (10.68 ± 3.57 vs. 13.24 ± 3.64; *p* < 0.05), which was an expected finding.

During pregnancy, the immune system turns to a Th2 response to induce tolerance in the fetus, AD itself being a Th2-mediated disease [18]. This change makes it the most frequent dermatosis in pregnancy, and flares typically occur in the second or third trimester. There are authors who speak of atopic eruption of pregnancy (AEP) for those cases of de novo appearance in pregnancy and that it is clinically and histologically indistinguishable from classic AD [12]. Regarding classical therapy, there is sufficient consensus to consider topical corticosteroids and phototherapy (NB-UV) as first-line therapies in the planning periods of pregnancy, pregnancy, and breastfeeding. Topical calcineurin inhibitors, cyclosporine, and azathioprine may be reasonable options, but with a lower degree of evidence, mycophenolate mofetil and methotrexate would be formally contraindicated for their use [19]. With regard to the new therapies, the involvement of IL4, IL13, and the JAK-STAT pathway in their pathogenesis makes us consider the positioning of these therapies in pregnancy and lactation. In relation to JAK inhibitors, the data sheet of all of them recommends their interruption 1 month before pregnancy and evaluates their reintroduction according to the clinical situation of the mother in lactation. Dupilumab has begun to report evidence of no effect on pregnancy and delivery without unexpected complications. In May 2019, a retrospective study began to describe the adverse effects of dupilumab during pregnancy (NCT03936335), whose data are expected to be completed in July 2027 [20].

There are no specific studies that assess the impact of AD in this important life stage for women, and in this sense, we believe that it is very important to have tools that allow us to explore all the possible areas involved. Johansen et al. measured awareness and expectations around family planning and pregnancy among Danish patients with chronic inflammatory disease of the skin or joints. They concluded that patients expressed a feeling of limited access to information and have concerns that affect key decisions about FPP, existing a need for improved and more standardized FPP information for patients with chronic inflammatory diseases [7].

Our study has some limitations that should be taken into account. On the one hand, the patients who have been included belong to specialized hospitals. On the other hand, we currently have very effective treatments that achieve high responses, so the level of severity measured by scales such as EASI may not be representative of the overall severity of the disease. Therefore, the study population may not be representative of the general population in terms of severity, socioeconomic status, education, and access to health care resources, and the results of this study may not be extrapolated to the general population.

## 5. Conclusions

In summary, the final objective of this questionnaire was to improve the results in this area. We would like to remark on the importance of the test itself and its reliability due to the high overall Alpha Cronbach of 0.94, especially in the first four dimensions. Preconception counseling should be offered to all patients of reproductive age, regardless of treatment. A well-informed patient is more likely to achieve the goals established. Proper planning of pregnancy is essential, and recommending and achieving therapeutic goals with drugs that are safe from the gestational point of view and for the fetus. Our findings confirm that there are two variables that present a greater difference in the family planning of women with AD, having been a mother before, and the use of systemic or biological drugs. Although we cannot act on these variables, we have identified a subgroup of patients where informative interventions can have more impact. Furthermore, the impact on the sexual life of women with AD has been highlighted, being another aspect where intervention should be made. There is no doubt that the probability of a successful pregnancy will also be greater if it is performed in a multidisciplinary care context, working as a team, with the identification and understanding of the possible particular risks for an individual patient. In fact, this validated questionnaire could be applied to AD patients who are treated in hospitals and thus benefit from possible interventions.

## Figures and Tables

**Table 1:** Sociodemographic characteristics of our sample.

	AD Cases (n = 75)	Controls (n = 75)
Age (Years) Average (SD)		
	29.72 (7.68)	30.32 (7.51)
Academic level (n,%) Without studies or unfinished Primary studies Medium professional education Higher professional education Baccalaureate University studies or equivalent		
	2 (2.66%)	0
	0	1 (1.33%)
	6 (8.00%)	12 (16.00%)
	18 (24.00%)	12 (16.00%)
	12 (16.00%)	14 (18.67%)
	37 (49.33%)	36 (48.00%)
Place of residence Municipality < 20,000 Municipality 20,000–50,000 Municipality > 50,000		
	21 (28.00%)	18 (24.00%)
	35 (46.67%)	31 (41.33%)
	19 (25.33%)	26 (34.67%)
Civil status Married Single/separated Live as a couple		
	31 (41.33%)	35 (46.67%)
	25 (33.33%)	17 (22.67%)
	19 (25.33%)	23 (30.67%)
She has children? Yes (n,%)	47 (62.67%)	51 (68.0%)
Clinical variables	EASI (n, %) ≤7: 65 (86.67%) 7–21: 7 (9.33%) ≥21: 3 (4.0%)	Reason for consultation (n, %) Acne: 7 (9.33%) Nevus control: 40 (53.33%) Seborrheic dermatitis: 10 (13.33%) Effluvium: 5 (6.67%) Rosacea: 6 (8.00%) Others: 7 (9.33%)
	DLQI (n, %) ≤3: 37 (49.33%) 3–10: 33 (44.00%) ≥10: 5 (6.67%)	
	DA years of evolution (mean, SD): 13.14 (8.33)	
	Systemic treatment %: 45 (60.0%)	
	Biological treatment %: 2 (2.67%)	

**Table 2:** Final version of the questionnaire.

**Spanish version used for validation** ¿Qué grado de acuerdo o desacuerdo tiene con las distintas afirmaciones? 1. Me siento o me, he sentido incómoda o cohibida debido a mi enfermedad. 2. Mi enfermedad afecta a mi vida sexual. 3. Tener descendencia es muy importante para mi. 4. Tener descendencia es un tema que me preocupa. 5. Considero que mi enfermedad puede empeorar con el embarazo. 6. Considero that my embarazo puede sufrir complicaciones por mi enfermedad. 7. La idea de que mis hijos puedan heredar mi enfermedad limita mi deseo de tener descendencia. 8. La planificación familiar (tener descendencia) es un tema que se aborda en consulta. 9. Antes de intentar concebir, he abordado con mi dermatólogo/a el tema de querer tener descendencia. 10. Antes de intentar concebir, he elaborado junto con mi dermatólogo/a un plan de tratamiento sobre los medicamentos que debo o no debo tomar. 11. La idea de que mi enfermedad empeore al tener que retirar o cambiar el fármaco antes del embarazo me preocupa. 12. Soy consciente de la necesidad de tener la actividad de mi enfermedad controlada antes del embarazo. 13. Soy consciente de la necesidad de tener la actividad de mi enfermedad controlada durante el embarazo. 14. La idea de que mi enfermedad empeore al tener que retirar o cambiar el fármaco durante del embarazo me preocupa. 15. La idea de que los tratamientos que reciba por mi enfermedad puedan llegar a mi bebé me preocupa. 16. El dermatólogo/a me ha informado de las opciones sobre lactancia materna. 17. La idea de que mi enfermedad empeore al tener que retirar o cambiar el fármaco durante la lactancia materna me preocupa. 18. Considero que mi enfermedad puede limitarme para cuidar un hijo adecuadamente. 19. Si recibiera más información por parte de mi médico/a en este sentido, me ayudaría a replantearme mi deseo de tener hijos. 20. He consultado fuentes de información externas (web, revistas, televisión…) sobre este tema de planificación familiar/embarazo/lactancia materna en pacientes con mi enfermedad. 21. Si pudiera tomar decisiones de forma conjunta con mi dermatólogo/a me daría seguridad para plantearme descendencia. 22. No tengo en cuenta los tratamientos que realizo para la planificación familiar. 23. Creo que en mi planificación familiar no debe interferir las recomendaciones del dermatólogo/a. *Opciones de respuesta para cada pregunta: Muy en desacuerdo; En desacuerdo; Ni de acuerdo ni en desacuerdo; De acuerdo; Muy de acuerdo*
**English translation performed for this article (not validated)** What degree of agreement or disagreement do you have with the different statements? 1. I feel or have felt uncomfortable or self-conscious due to my illness. 2. My disease affects my sexual life. 3. Having children is very important to me. 4. Having offspring is an issue that worries me. 5. I think my illness can worsen with pregnancy. 6. I consider that my pregnancy may suffer complications due to my illness. 7. The idea that my children may inherit my disease limits my desire to have children. 8. Family planning (having offspring) is a topic that is addressed in consultation. 9. Before trying to conceive, I discussed with my dermatologist the question of wanting to have children. 10. Before trying to conceive, I developed a treatment plan with my dermatologist about the medications I should or should not take. 11. The idea that my disease will worsen when I have to withdraw or change the drug before pregnancy worries me 12. I am aware of the need to control my disease activity before pregnancy. 13. I am aware of the need to control my disease activity during pregnancy. 14. The idea that my disease will worsen when I have to withdraw or change the drug during pregnancy worries me. 15. The idea that the treatments I receive for my illness could reach my baby worries me. 16. The dermatologist has informed me about the options for breastfeeding. 17. The idea that my disease will worsen when I have to withdraw or change the drug during breastfeeding worries me. 18. I consider that my illness may limit me to adequately caring for a child. 19. If I received more information from my doctor on this, it would help me to rethink my desire to have children. 20. I have consulted external sources of information (web, magazines, television, etc.) on this topic of family planning/pregnancy/breastfeeding in patients with my disease. 21. If I could make decisions together with my dermatologist, it would give me the security of considering my descendants. 22. I do not take into account the treatments I perform for family planning. 23. I believe that dermatologist recommendations should not interfere with my family planning. Response options for each question: Strongly disagree; In disagreement; Neither agree nor disagree; Agree; Strongly agree

**Table 3:** Dimensions of the questionnaire.

Dimension	Questionnaire Items	Cronbach’s Alpha
“Pregnancy”	5, 6, 12, 13, 14, 15	0.95
“Conception”	7, 8, 9, 10, 11	0.93
“Concern-information”	3, 4, 19, 20	0.82
“Breastfeeding”	16, 17, 18	0.81
“Sexual life”	1, 2	0.79
“Family Planning”	21, 22 *, 23 *	0.67

* Items written in negation that have been reconverted for the EFA.

**Table 4:** Scores obtained in the questionnaire.

Scale	Item	AD Group			Control Group		
		Mean	SD	Range	Mean	SD	Range
“Pregnancy”	Item 5	3.89	1.36	1–5	2.27	1.50	1–5
	Item 6	3.56	1.47	1–5	1.89	1.19	1–5
	Item 12	4.23	1.07	1–5	2.79	1.74	1–5
	Item 13	4.31	0.99	1–5	2.96	1.77	1–5
	Item 14	4.30	0.96	1–5	2.95	1.72	1–5
	Item 15	4.31	1.00	1–5	3.21	1.74	1–5
	*Scale*	*4.09*	*1.19*	*6–30*	*2.58*	*1.64*	*6–30*
“Conception”	Item 7	3.57	1.49	1–5	2.08	1.24	1–5
	Item 8	4.08	1.22	1–5	2.06	1.48	1–5
	Item 9	3.98	1.39	1–5	1.58	1.04	1–4
	Item 10	3.77	1.49	1–5	1.52	0.98	1–4
	Item 11	4.01	1.29	1–5	1.91	1.35	1–5
	*Scale*	*3.88*	*1.38*	*5–25*	*1.86*	*1.26*	*5–23*
“Concern-information”	Item 3	3.85	1.22	1–5	3.86	1.14	1–5
	Item 4	3.49	1.40	1–5	3.61	1.25	1–5
	Item 19	2.16	1.00	1–5	2.22	1.25	1–5
	Item 20	2.49	1.24	1–5	2.30	1.38	1–5
	*Scale*	*3.01*	*1.40*	*4–20*	*3.06*	*1.45*	*4–20*
“Lactation”	Item 16	2.13	1.24	1–5	1.08	0.28	1–2
	Item 17	2.65	1.55	1–5	1.24	0.60	1–4
	Item 18	2.26	1.34	1–5	1.33	0.73	1–3
	*Scale*	*2.34*	*1.40*	*3–15*	*1.23*	*0.59*	*3–9*
“Sex life”	Item 1	3.47	1.44	1–5	3.45	1.48	1–5
	Item 2	3.03	1.36	1–5	2.47	1.44	1–5
	*Scale*	*3.25*	*1.41*	*12–10*	*2.96*	*1.53*	*2–10*
“Family planning”	Item 21	3.20	1.23	1–5	2.76	1.54	1–5
	Item 22	2.58	1.21	1–5	2.44	1.48	1–5
	Item 23	3.10	1.23	1–5	3.75	1.35	1–5
	*Scale*	*2.96*	*1.25*	*3–9*	*3.02*	*1.56*	*3–9*
*Global*		*78.43*	*1.46*	*23–115*	*55.75*	*1.55*	*23–107*

**Table 5:** Relationship between scales and variables in the AD group.

Variable	Cut-Off	n	Factor 1		Factor 2		Factor 3		Factor 4		Factor 5		Factor 6		Escala Global	
			Mean ± SD	*p*	Mean ± SD	*p*	Mean ± SD	*p*	Mean ± SD	*p*	Mean ± SD	*p*	Mean ± SD	*p*	Mean ± SD	*p*
Age	<30 >30	42 33	24.26 ± 7.06 24.97 ± 3.57	0.616	19.89 ± 5.57 19.42 ± 4.84	0.730	11.72 ± 4.18 12.73 ± 3.33	0.276	6.38 ± 3.08 7.67 ± 3.75	0.137	6.56 ± 3.97 6.41 ± 2.20	0.809	8.40 ± 2.92 9.55 ± 2.63	0.100	75.79 ± 21.04 53.42 ± 11.39	0.112
Educational level	NU U	38 37	25.34 ± 5.06 23.76 ± 6.41	0.260	19.17 ± 5.76 20.18 ± 4.77	0.447	11.91 ± 3.85 12.50 ± 3.79	0.526	6.31 ± 3.36 7.53 ± 3.39	0.154	7.027 ± 2.50 5.95 ± 2.69	0.075	8.94 ± 2.65 8.94 ± 3.04	1.000	79.62 ± 17.96 78.28 ± 18.27	0.778
Years duration AD	<13 >13	46 29	23.79 ± 5.84 25.85 ± 5.54	0.153	19.55 ± 5.22 20.00 ± 5.40	0.750	11.67 ± 3.63 13.26 ± 3.99	0.103	6.87 ± 3.33 7.05 ± 3.69	0.844	6.00 ± 2.42 7.32 ± 2.80	**0.035**	9.33 ± 2.67 8.25 ± 3.03	0.135	76.39 ± 16.82 84.67 ± 19.58	0.104
EASI	<=7 >7	67 8	24.49 ± 6.07 25.29 ± 1.80	0.442	19.71 ± 5.19 19.60 ± 6.54	0.966	12.19 ± 3.89 12.33 ± 3.08	0.931	6.78 ± 3.46 8.60 ± 2.30	0.255	6.31 ± 2.71 8.00 ± 1.07	**0.003**	8.82 ± 2.73 10.17 ± 3.76	0.268	78.36 ± 18.34 85.20 ± 13.29	0.421
DLQI	<=3 >3	39 36	25.72 ± 5.15 23.30 ± 6.22	0.082	20.33 ± 5.24 19.00 ± 5.25	0.317	12.08 ± 3.82 12.35 ± 3.84	0.767	6.88 ± 4.17 6.97 ± 2.34	0.920	5.70 ± 2.82 7.40 ± 2.09	**0.005**	8.94 ± 2.77 8.94 ± 2.93	0.996	79.93 ± 17.72 77.89 ± 18.49	0.670
Children	0 =>1	28 47	21.19 ± 7.11 26.60 ± 3.55	**0.001**	16.74 ± 5.62 21.40 ± 4.22	**0.000**	9.46 ± 3.61 13.67 ± 3.04	**0.000**	5.83 ± 2.69 7.54 ± 3.64	**0.037**	6.11 ± 2.56 6.72 ± 2.68	0.330	6.88 ± 2.54 10.12 ± 2.27	**0.000**	65.35 ± 18.87 87.89 ± 10.18	**0.000**
Systemic or biologic treatment	No Yes	28 47	23.23 ± 6.11 25.37 ± 5.48	0.137	18.42 ± 5.25 20.59 ± 5.12	0.106	10.68 ± 3.57 13.24 ± 3.64	**0.005**	5.25 ± 3.00 8.22 ± 3.16	**0.000**	5.90 ± 2.83 6.89 ± 2.74	0.112	8.58 ± 2.82 9.20 ± 2.85	**0.405**	72.15 ± 18.71 84.47 ± 15.52	**0.008**

U: University studies; NU: Non-university studies. Bold numbers mean statistical significance.

## Data Availability

The data presented in this study are available on request from the corresponding author. The data are not publicly available due to privacy.

## Supplementary Materials

The following supporting information can be downloaded at: https://www.mdpi.com/article/10.3390/ijerph191710753/s1, Table S1. Regression analysis of global score and variables in the AD group.
