# Supplementary material for: Validation of a Questionnaire to Assess the Perception of Women with Atopic Dermatitis in Family Planning
Source: Int J Environ Res Public Health. 2022 Aug 29;19(17):10753. doi: 10.3390/ijerph191710753 (PMC9517831; doi:10.3390/ijerph191710753)
Supplement: Supplementary file 1 [file ijerph-19-10753-s001.zip › ijerph-1880405-supplementary.pdf]

Table S1. Regression analysis of global score and variables in the AD group

Variable	β	p	R^2
Age >30 years	-,069	0.555	0.436
University studies	-0.022	0.848	
Years duration DA >13 years	0.045	0.673	
EASI > 7	0.001	0.992	
DLQI >3	-0.088	0.394	
Has Children	,574	**0.000**	
Sistemic or biologic treatment	,234	**0.027**	

β: standardized regression coefcient; p: p-value
